# Odor Identification Across Time in Mutation Carriers and Non-Carriers in Autosomal-Dominant Alzheimer’s Disease

**DOI:** 10.3233/JAD-230618

**Published:** 2024-01-16

**Authors:** Ove Almkvist, Maria Larsson, Caroline Graff

**Affiliations:** a Division of Clinical Geriatrics, Department of Neurobiology, Care Sciences and Society, Karolinska Institutet, Stockholm, Sweden; b Division of Neurogeriatrics, Department of Neurobiology, Care Sciences and Society, Karolinska Institutet, Stockholm, Sweden; c Gösta Ekman Laboratories, Department of Psychology, Stockholm University, Stockholm, Sweden; d Theme Inflammation and Ageing, Karolinska University Hospital, Stockholm, Sweden; e Department of Psychology, Stockholm University, Stockholm, Sweden

**Keywords:** Alzheimer’s disease, autosomal-dominant Alzheimer’s disease, cognition, mutation carriers, non-carriers, odor identification

## Abstract

**Background:**

Impaired odor identification is a characteristic of sporadic Alzheimer’sdisease(AD), but its presence in autosomal-dominantAD (adAD) remains uncertain.

**Objective:**

To investigate odor identification ability in mutation carriers (MC) and non-carriers (NC) of adAD in relation to years to estimated clinical onset clinical onset (YECO) of disease.

**Methods:**

Participants from six families with autosomal-dominant mutations (*APP* Swedish, *APP* Arctic, and *PSEN1* mutations) included 20 MC and 20 NC. The groups were comparable in age, gender, education, number of *APOE* ɛ4 alleles, and YECO, but differed in global cognition (Mini-Mental State Examination). The MC group included individuals in asymptomatic, symptomatic cognitively unimpaired, mild cognitive impairment, and dementia stages of disease, spanning approximately 40 years of the AD continuum. All NC were asymptomatic. Olfactory function was assessed by means of free and cued identification of common odors summarized as total identification.

**Results:**

MC performed poorer than NC in free and total identification. Four MC and none of the NC were anosmic. Olfactory functions in MC and NC were significantly and inversely related to time course (YECO) for both free and total identification. The decline in free identification began approximately 10 years prior to the estimated clinical onset of AD in MC. Odor identification proficiency was associated with episodic memory and executive function in MC and NC.

**Conclusions:**

Impaired odor identification is present well before the clinical diagnosis of AD in MC and is associated with disease progression. Odor identification ability may be a useful early biomarker for adAD.

## INTRODUCTION

It is well established that olfactory function is impaired in various neurodegenerative diseases including Alzheimer’s disease (AD) [1–3], vascular dementia [4], frontotemporal dementia, Lewy body disease, and Parkinson’s disease [5]. Research indicates that olfactory dysfunction is prevalent in preclinical stages of sporadic AD (sAD), represented by mild cognitive impairment (MCI) [6] and deficits are typically more pronounced than those observed in normal aging [1, 7, 8].

Despite this knowledge, it remains unclear when olfactory impairment begins in the development of AD. To address this gap, researchers have studied autosomal-dominant families with AD (adAD). In individuals who carry a mutation in the *APP* and *PSEN1* genes in adAD, it is possible to calculate the time point in years of disease progression. One study of the Colombian *PSEN1*_E280A_ mutation family showed that mutation carriers (MC) did not differ from non-carriers (NC) in cued odor identification, although they differed in cognition and cerebrospinal fluid biomarkers of AD [9]. However, olfactory dysfunction was more prevalent among older MC than among older NC, suggesting that odor impairment may be an early marker of brain pathology and future risk of dementia [9]. As only one mutation in the *PSEN1* gene was investigated in the Colombian family, further research is required to explore olfactory function not only by means of cued identification but also with the use of free, cued, and total (summary of free and cued) identification of odors in families affected by different mutations in adAD.

The primary aim of the present study was to investigate free and total odor identification in MC, who ultimately develop AD, and in NC, who lack the mutation and the genetic risk to develop AD. MC and NC originated from two *APP* families and four *PSEN1*adAD families. The secondary aim was to investigate olfactory function in relation to a time scale expressed as years to expected clinical onset (YECO). The study covers the disease progression over four decades, including preclinical and clinical disease stages. The hypotheses were that MC and NC should differ in both free and total identification, due to the presence or absence of an adAD mutation and that both olfactory measures were negatively influenced by disease progression as indicated by YECO. Further, the relationship between olfactory function and cognitive abilities were investigated in MC. The expectation was that olfactory function is primarily related cognitive functions that are sensitive to decline in cognition in AD. By studying families affected by different mutations in adAD genes, this research will provide insights into the time-course of olfactory impairment in adAD.

## MATERIALS AND METHODS

### Participants

Adults from six families, in which a mutation predisposing for adAD had been identified, were invited to research visits at the Memory Clinic, Karolinska University Hospital, Stockholm. Two families had a mutation in the amyloid precursor protein gene (*APP*), the Swedish mutation (*APP*_SWE_) [10], and the Arctic mutation (*APP*_ARC_) [11]. Four families had a mutation in the presenilin 1 gene (*PSEN1*): *PSEN1*_H163Y_ [12], *PSEN1*_M146V_ [13], *PSEN1*_I143T_ [14], and *PSEN1*_L232P_ [15]. The study included 20 MC, whose clinical characteristics were consistent with typical AD [11–16] and 20 cognitively unimpaired NC serving as healthy controls. The number of individuals with a mutation in each family is not disclosed to maintain confidentiality of their mutation status.

### Diagnosis of cognitive impairment and AD

Based on the examinations at the research visits, 10 out of 20 MC were asymptomatic and without objective impairment, four MC showed minor objective cognitive changes that did not meet the criteria for MCI or dementia, and they did not report any relevant subjective symptoms required for the diagnosis of subjective cognitive impairment [17]. Two MC were diagnosed with MCI [18], and four MC were diagnosed with dementia according to DSM-IV [19] and AD according to NINCDS-ARDRA criteria (including both probable and possible AD) [20]. None of the NC were diagnosed with dementia or MCI, and none of them exhibited any symptoms associated with cognitive deficits. One NC had selective difficulties that affected the visuospatial domain exclusively as observed across three tests (Block Design, Rey-Osterrieth copy and retention) and not in other tests. The deficit had been prevalent during the whole life span and was considered as a hereditary spatial disorientation syndrome without any connection to AD or other brain disease [21].

### Procedure

Each participant underwent a standardized comprehensive clinical examination that included interviews with both the participant and a close informant. The examination assessed the participant’s somatic, neurologic, cognitive, and psychiatric status. Blood and cerebrospinal fluid were sampled and analyzed for *APOE* status, AD biomarkers (Aβ_42_, total-tau, and phosphorylated tau (p-tau)). The brain was scanned using magnetic resonance imaging (MRI) to evaluate the degree of atrophy, white matter changes, and other brain abnormalities. In addition, olfactory function was assessed, and the results were not used in clinical evaluation of participants. The participants and all research personnel were blinded to the mutation status.

### Years to expected clinical onset (YECO)

For every individual, the time of disease progression was defined by calculating the number of YECO, defined as the age of the individual minus the expected family-specific age at AD diagnosis [22, 23]. The clinical onset was defined as the age at which the first relevant symptoms appeared [22, 23]. The mean age 6 of clinical onset for each mutation was calculated from previous family history as described in medical records and is relatively fixed and specific for each mutation [24]. It is noteworthy that YECO seems to be a reliable and valid measure for monitoring the disease course and that expected clinical onset is usually strongly associated with observed clinical onset in adAD [22–24]. The age at the observed clinical onset varies depending on the mutation, ranging from the thirties in *PSEN1* mutations: 36±2 years for *PSEN1*_I143T_ [14, 25], 36±3 years for *PSEN1*_M146V_ [13], 37±1 years for *PSEN1*_L232P_ [15] and to the fifties in one *PSEN1* mutation 52±6 years for *PSEN1*_H163Y_ [12, 25] and two *APP* mutations 54±5 years for *APP*_SWE_, [16, 25], and 56±4 years for *APP*_ARC_ [11, 25, 26].

The YECO is time-related in relation to the expected clinical onset and is not collinear with the participant’s age. It is a widely used measure as shown in previous research [22, 23]. The preclinical stage is defined as YECO < 0,while the clinical stages are defined as YECO≥0. In addition, cognitive reserve, often measured by years of formal education, was also evaluated as a potential predictor of disease-related cognitive decline [27].

### Genotyping

The genotyping of adAD mutations and *APOE* has been described in detail in a previous publication [26].

### Assessment of cognitive function

The study employed a comprehensive battery of tests to assess cognitive function in participants. Current global cognitive function was evaluated by aggregating the results from the Swedish version of Wechsler Adult Intelligence Scale Revised [28, 29], which provides a measure of overall cognitive ability. To assess premorbid cognitive function, the aggregated results from the Swedish Irregularly Spelled Words test [30, 31] and the Swedish Lexical Decision test [32] were used. Global cognitive deterioration was quantified by computing the difference between current global and premorbid cognition scores; all scales were expressed with the same metric standardized in normal aging (IQ scale, M±SD, 100±15).

In addition to the global assessment, cognitive function was evaluated in five domains: verbal abilities, visuospatial abilities, executive function, episodic memory, and attention. The domains were assessed by using the Similarities, Block Design, and Digit Symbol tests from the Swedish version of Wechsler Adult Intelligence Scale Revised [28, 29],as well as the RAVL learning (episodic memory) and Trail Making part A (attention) tests [33].The raw scores were converted to z-scores using a reference group of healthy adults at Karolinska University Hospital at Huddinge [34]. Two MC were unable to complete the neuropsychological assessment due to marked cognitive impairment causing some missing data when cognition performance is analyzed. Notably, all cognitive and olfactory function assessments were conducted by the same clinical psychologist, which enhances consistency in the data collection process.

### Assessment of olfactory function

Olfactory function was assessed using the Sniffin’ Stickstest (Burghart Medical Technology, Wedel, Germany), a widely used norm-referenced test with good psychometric data [35–37]. Participants are presented with 16 felt tip-pens containing common everyday odors: anise, apple, banana, clove, coffee, cinnamon, fish, garlic, lemon, leather, licorice, mint, orange, pineapple, rose, and turpentine. For each odor, participants are asked to identify the odor by providing a verbal descriptor (free identification). A liberal criterion was used for scoring (e.g., citrus fruit for orange, Christmas or dentist for clove). Proportion correct was computed by dividing the number of correctly identified odors by 16. If participants failed to retrieve a correct name, they were presented with 4 different written response alternatives, 1 target and 3 foils, and were instructed to choose the label that best matched the specific odor (cued identification). The total identification score was the summarized score of free and cued identification divided by the total number of odors presented. Anosmia, hyposmia, and normosmia were defined based on the total identification score [37–40].

The free identification task required the participants to perceive the presented odor, search and retrieve the appropriate name from semantic memory and provide a response. In contrast, cued identification relieved participants from having to retrieve the name from memory, as the correct odor name was presented among the response alternatives. In brief, olfactory function was assessed based on the perceptual, cognitive, and mnemonic processes involved in identifying odors [7].

### Statistics

In general, individuals in the two groups were randomly sampled and observations were independent. Outliers may exist due to latent or manifest disease in the MC group that may violate normal distributions. The same argument holds for homogeneity of variances in the MC group. Difference between MC and NC groups were analyzed by *t*-tests for continuous variables and χ^2^ for categorical variables (Tables 1, 2, and 5). The assumptions (normality and homoscedasticity) for these statistical analyses were not violated except for YECO and MMSE. However, the MMSE was significant after correction. Associations between continuous variables were analyzed with Pearson correlations (Tables 3, 5, and 6). The assumptions of interval data were fulfilled for all variables. The assumptions (normality and homoscedasticity) were not violated in NC, but in MC. The regression analyses of odor performance as criterion were performed separately in free and total identification with mutation status (MC versus NC) and timeline (YECO) as independent variables (Table 4). The number of independent variables were limited to two variables given the small size (*n* = 40). There was no violation of normal distributions, 9 homoscedasticity, linearity, or multicollinearity for free odor identification, while objections regarding linearity were present for total odor identification that expelled firm conclusions for total identification. Missing data occurred for three MC individuals in the correlation analyses regarding Similarities, RAVL and Digit Symbol (Table 6).

### Ethics

When the adAD project started, all potential participants were informed about their risk of inheriting AD. All individuals who accepted to participate in the project received genetic counseling in connection with the study. All subjects provided written informed consent to participate in the present study and other separate studies within the adAD project. The study was approved by the Ethics Committee of and conducted according to the declaration Helsinki and subsequent revisions (2006/901-31/7; 2022-06565-02).

## RESULTS

The demographics (age, sex, education), clinical characteristics (YECO, MMSE, *APOE* ɛ4 frequency), and global cognitive function (current, premorbid and change) for the MC and NC groups are presented in Table 1 showing M±SD, 95% CI, *p*-value and effect size of group differences. The MC group performed significantly poorer in the MMSE, which measures global cognitive function [41], (*t*(36) = 2.45, *p* < 0.05), and experienced a more pronounced decline in IQ from premorbid to current cognitive function compared to the NCgroup (*t*(36) = 2.42, *p* < 0.05).There were no statistical differences between the two groups inage, gender distribution, YECO, years of education, premorbid IQ, current IQ, and number of *APOE* ɛ4 alleles. Notably, the YECO ranged from –33 (earliest) to +8 (latest) for the MC group and NC varied in range of YECO from –28 (earliest) to +11 (latest) and accordingly about 40 years of disease course was covered.

**Table 1:** Demographic and clinical characteristics and group differences between mutation carriers (MC) and non-carriers (NC) in autosomal-dominant Alzheimer’s disease with *p*-value and effect size (Cohen’s *d*)

	Group	
	MC (*n* = 20)	NC (*n* = 20)	*p*	Cohen’s *d*
Age, y	47.5±12.8	44.3±12.5	ns	0.26
Sex (female/male), proportion	11/9	6/14	ns	0.51
Education, y	12.6±2.0	12.0±2.2	ns	0.30
YECO, y	–4.3±12.0	–6.7±10.3	ns	0.22
MMSE, score	26.3±5.1	29.2±1.0	< 0.05	0.80
*APOE* ɛ4, proportion	0.44±0.51	0.33±0.59	ns	0.34
Premorbid global cognition, IQ	107.5±8.7	102.4±6.9	ns	0.63
Current global cognition, IQ	85.9±22.6	96.7±17.3	ns	0.54
Current-Premorbid, IQ	–19.3±19.59	–3.2±14.2	< 0.05	0.92

### Olfactory function in relation to mutation status

The results on free and total odor identification for MC and NC groups with *p*-values and effect size (Cohen’s *d*) are presented in Table 2. The MC group performed clearly poorer than the NC group both in free (*t*(36) = 3.43, *p* < 0.01, Cohen’s *d* = 1.14) and total odor (*t*(36) = 2.82, *p* < 0.01, Cohen’s *d* = 1.01) identification. Further, four MC individuals were anosmic according to common criteria for total odor identification [36–39], while no NC individual was anosmic. In contrast, 18 NC were evaluated as normal in total odor identification, while 12 MC individuals performed normally in odor identification. In summary, these results regarding the first aim, indicate that olfactory function is influenced by mutation status.

**Table 2:** Free and total odor identification in mutation carriers (MC) and non-carriers (NC) in autosomal-dominant Alzheimer’s disease with *p*-value and effect size (Cohen’s *d*)

	Group	
Odor identification	MC (*n* = 20)	NC (*n* = 20)	*p*	Cohen’s *d*
Free	0.197±0.181	0.403±0.180	< 0.01	1.01
Total	0.625±0.314	0.869±0.130	< 0.001	1.88

### Olfactory function in relation to time scale

The correlation coefficients between free and total odor identification versus the time scale (YECO) for MC and NC are presented in Table 3. The pattern of associations demonstrated decline in olfaction linked to the time scale that was marked and significant in free identification for MC(*r*^2^ = 0.59, *p* < 0.001), but not significant in NC. The associations for total identification were significant and less marked both for MC (*r*^2^ = 0.28, *p* < 0.05) and NC (*r*^2^ = 0.25, *p*≤0.05). Regarding the second aim, the overall findings indicate that olfactory functions are influenced by time.

**Table 3:** Correlation coefficients between olfactory function (free and total identification) versus years to estimated clinical onset (YECO) in mutation-carriers (MC) and non-carriers (NC). Significant correlations are bolded

	Odor identification			
	Free		Total	
Time measure	MC	NC	MC	NC
YECO, y	–0.77^ ***^	–0.40^ns^	–0.53^*^	–0.49^*^

^*^p < 0.05; ^**^p < 0.01; ^***^p < 0.001.

### Olfactory function in relation to mutation status and YECO

The relationship between free odor identification versus time (YECO) and mutation status as independent variables showed that the model was significant, *F*(2, 37) = 22.64, *r* = 0.74, *p* < 0.001, with the two predictors: mutation status (*B* = –1.09, *beta* = –0.47^***^, 95% *CI*:–1.61 to 0.57), and YECO (*B* = –0.06, *beta* = –0.52^***^, 95% *CI*: –0.08 to –0.03), see Table 4, upper panel.The relationship between total odor identification versus time (YECO) and mutation status as independent variables showed that the model was significant as well, *F*(2, 37) = 12.55, *r* = 0.64, *p* < 0.001, with the two predictors: mutation status (*B* = –1.69, *beta* = –0.41^***^, 95% CI: –1.62 to 0.57) and YECO (*B* = –0.06, *beta* = –0.52^***^, 95% *CI*: –0.08 to –0.03), see Table 4, lower panel.

**Table 4:** Linear regression analyses with olfactory function as dependent variable and mutation status (MC versus NC) and time (YECO) as independent variables and in free (upper panel) and in total identification of odors (lower panel) in proportion correct

Free identification
Free identification mutation	B		β
Mutation	*r* = –0.53^***^		–1.09	–0.47^***^
YECO	*r* = –0.57^***^	*r* = +0.11^ns^	–0.055	–0.523^***^
	Intercept = –0.366
Free identification mutation	YECO		*r*^2^ = 0.55	
Mean	–0.61	0.50	–5.48	adjusted *r*^2^ = 0.53
SD 1.17	0.51	11.10
Total identification
Total identification mutation	B		β
Mutation	*r* = –0.46^**^		–1.69	–0.415^***^
YECO	*r* = –0.48^**^	*r* = +0.11^**^	–0.082	–0.440^***^
	Intercept = –0.366
Total identification mutation	YECO		*r*^2^ = 0.40	
Mean	–0.94	0.50	–5.48	adjusted *r*^2^ = 0.37
SD 2.06	0.51	11.10

**Fig. 1:**
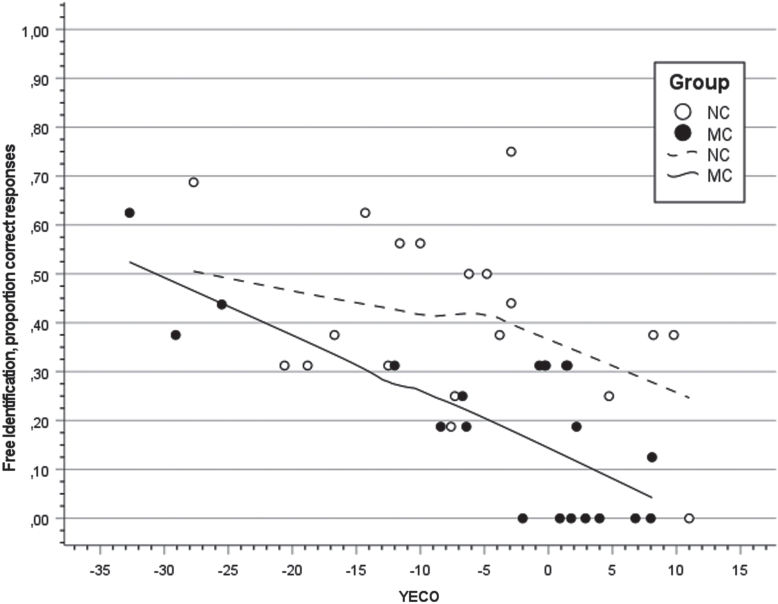
Scatter plot and regression lines with locally estimated smoothing for the proportion of free odor identification in relation to Years to Estimated Clinical Onset (YECO) inmutation carriers (MC; filled black) and non-carriers (NC; unfilled).

Figure 1 presents a scatter plot of regression with free odor identification in MC and NC overtime (YECO) as independent variable using locally estimated scatter plot smoothing (LOESS) procedure for visualization of trajectories in MC and NC. In MC, observations indicated a linear regression and results supported this observation showing a significant linear relationship (*r* = 0.778, *F*(1,18) = 27.77, *p* < 0.001) and the regression was significantly different from zero (*B*: –0.006, *CI*: –0.263 to –0.110). The curvilinear regression (YECO^2^) was also significant (*r* = 0.702, *F*(1,18) = 17.47, *p* < 0.001) showing significant difference from zero (*B*: –0.007, *CI*: +0.003 to +0.010) NC.

### The effect of education and APOE ɛ4 on olfaction

The relationship between free and total odor identification and years of education and proportion of *APOE* ɛ4 in MC and NC is presented in Table 5. The association between total identification and years of education in NC was significant (*r* = 0.465, 95% *CI*: +0.028 to +0.753), but not in MC. Corresponding associations in free identification and education were not significant in MC and NC (*p*s > 0.1). The correlations between proportion of *APOE* ɛ4 and olfaction (free and total) were not significant in MC and NC (*p*s > 0.1).

**Table 5:** Correlation coefficients between olfactory function (free and total identification) versus years of education and presence of the *APOE* ɛ4 in mutation-carriers (MC) and non-carriers (NC). Significant correlations are bolded

	Identification			
	Free		Total	
Function/Measure	MC	NC	MC	NC
Education, y	–0.265^ns^	+0.364^ns^	+0.465^*^	+0.007^ns^
*APOE* ɛ4, prop.	+0.225^ns^	+0.442^ns^	+0.075^ns^	+0.276^ns^

^*^p < 0.05; ^**^p < 0.01; ^***^p < 0.001.

### Olfactory performance and cognitive functions in MC and NC

The correlations between free and total identification and separate cognitive functions in MC and NC are presented in Table 6. The pattern of associations was domain-specific showing significant positive associations between olfaction (free and total identification) versus episodic memory and executive function in both MC and NC. The strength of these associations was relatively equal in size. Current cognitive function and decline in cognition were significantly associated with total identification in MC (*p* < 0.05, 95% *CI*: 0.129 to 0.815; *p* < 0.05; 95% *CI*: 0.086 to 0.825; respectively). In addition, there was a significant association between visuospatial performance and total identification in MC (*r* = 0.578, *p* < 0.01, *CI*: 0.168 to 0.818).

**Table 6:** Correlation coefficients for mutation carriers (MC) and non-carriers (NC)between global current and premorbid cognitivefunction and cognitive decline (current-premorbid) and five cognitive domains for free and total identification. Significant correlations are bolded

	Identification			
	Free		Total	
Function/Measure	MC	NC	MC	NC
Premorbid cognition (IQ)	–0.02	0.13	–0.04	0.44
Current cognition (IQ)	0.36	0.22	0.56^*^	0.16
Decline in cognition (IQ)	0.33	–0.08	0.56^*^	–0.03
Verbal (Similarities)	0.23	0.36	0.37	0.13
Visuospatial (Block Design)	0.45	0.43	0.58^ **^	0.31
Episodic memory (RAVL)	0.57^*^	0.60^ **^	0.66^ **^	0.48^*^
Executive (Digit Symbol)	0.54^*^	0.66^ **^	0.46	0.59^ **^
Attention (TMT-A)	0.36	0.32	0.43	0.42

^*^p < 0.05; ^**^p < 0.01; ^***^p < 0.001.

### The decline in olfaction and cognition in relation progression of disease in MC

**Fig. 2:**
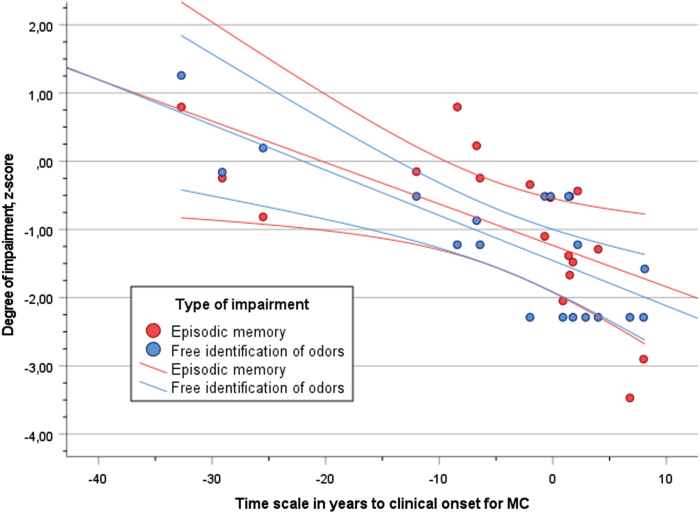
Scatter plot of performance (z-score) in episodic memory (RAVL, red dots) and free identification of odors (blue dots) in relation to time scale of years to clinical onset (YECO) for MC with 95% confidence interval.

**Fig. 3:**
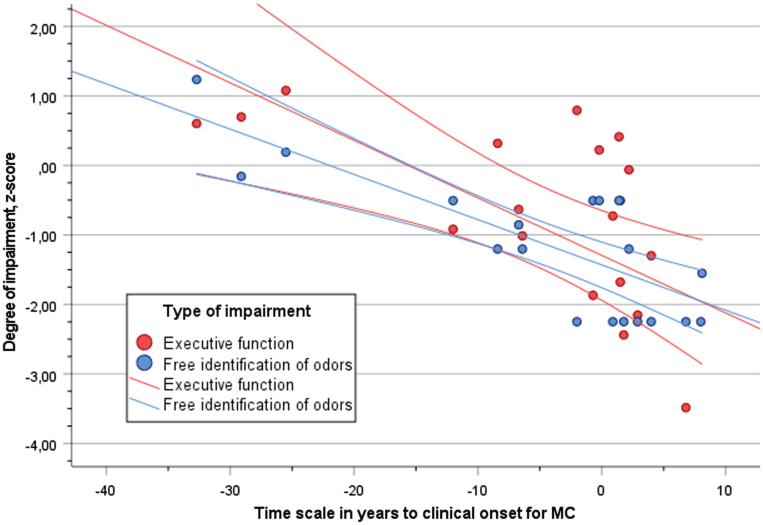
Scatter plot of performance (z-score) in executive function (Digit Symbol, red dots) and free identification of odors (blue dots) in relation to time scale of years to clinical onset (YECO) for MC with 95% confidence interval.

The degree of performance (z-score) in free odor identification and episodic memory(RAVL) across the time scale of disease progression (YECO) is visualized as linear relationships for both outcome abilities in Fig. 2. The strength of the associations between episodic memory and YECO (*r* = 0.64, *p* < 0.01) versus free identification and YECO (*r* = 0.77, *p* < 0.01) were not significantly different(*p* > 0.1).A similar pattern of results was obtained when degree of performance (z-score) in free odor identification and executive function (Digit Symbol) was investigated, see Fig. 3. A linear relationship with the time scale of disease progression (YECO) was shown for executive function (*r* = 0.61, *p* < 0.01).This association was not significantly different (*p* > 0.1) compared to the corresponding association for free identification (*r* = 0.77, *p* < 0.01).

## DISCUSSION

This study investigated free and total odor identification in adAD divided into MC and NC with comparable demographic characteristics. In addition, the relationship between olfactory function and years to the expected clinical onset of disease was analyzed. The third objective was to investigate the influence of cognition on olfactory function.

The results showed that MC were impaired in both free and total odor identification compared to NC. This result extends the result in a previous study on adAD with the *PSEN1*_E280A_ mutation [9] that showed impairments in cued odor identification about 5–10 years before expected onset. In addition, the present study included not only one specific mutation but four *PSEN1* mutations and two *APP* mutations. Furthermore, the difference in olfactory function in the present study was apparent as four MC were anosmic, while no NC was anosmic. The olfactory function across time decreased both in MC and NC, although more pronounced in MC than in NC. The trajectories of olfactory function in MC and NC began to diverge decades before the expected clinical onset in AD in both free and total identification. The difference between MC and NC became pronounced about 10 years before the expected clinical onset of AD. The decline in free odor identification was considered as linear in both MC and NC over the years, while total identification declined slowly in both groups until about 10 years before the estimated clinical onset at about 50 years of age. The difference between trajectories continued to increase when the cognitive impairment in MC increased.

The present results showing a difference in olfactory function in MC versus NC in adAD correspond with results in previous research in sAD and MCI showing impaired olfactory function [1, 6–8, 39–40]. The present results in NC from adAD families correspond with results in previous research showing some decline in normal aging too, although less pronounced than the decline in AD [1, 3, 7, 8]. The difference between MC and NC in the present study and between sAD and normal aging in previous research could be related to different etiologies. In AD, the disease is caused by pathological changes in the brain [42] and structural brain changes beginning in the medial temporal lobe [43, 44], while risk factors for cerebrovascular disease [45–47] or manifest cerebrovascular disease [4] is associated with olfactory dysfunction. In previous research, the ɛ4 allele of the *APOE* gene has been identified as risk factor for developing sporadic AD [48] and olfactory dysfunction [7, 46–49]. However, no such effect was obtained in the present study which may be due to lack of statistical power.

The difference in etiologies may explain the difference in the time-related decline in olfactory function exhibited by different rates of progression between MC and NC. In MC, the rate of decline was steeper than in NC, whereas the age-related decline in NC was less pronounced.

It is a typical finding that performance is poorer in free than total identification. It is unclear why the two indices of olfactory function show such different trajectories. However, to name an odor as required in free identification is a cognitively demanding task that involves search in memory and other mental processes [3, 7, 9, 49]. In this way, free odor identification requires multiple processes to be successful and some of these processes rely on cognitive abilities that are disturbed in AD. Consistent with previous research, olfactory function was associated with episodic memory and executive function in both MC and NC [7, 9, 49]. These relationships may have different causes in MC and NC, probably related to brain neurodegeneration in MC and probably related to manifest and incident cerebrovascular disease that are most common in NC [50].

A drawback of the present study relates to the small number of participants that increases the risk of type I error. Some statistical analyses may have violated assumptions because requirements of normality and homogeneity are not fulfilled in the MC group groups showing disease-related heterogeneity in outcome measures. Olfactory function across different mutations were not possible to compare due to sample size. However, compared to many other studies targeting AD, there is no diagnostic uncertainty. Furthermore, the use of a measure for disease progression, YECO (years to the clinical onset) made it possible to relate olfactory function directly to disease progression across the complete time span of disease evolution.

In conclusion, olfactory function differed clearly between MC and NC due to mutations in *APP* and *PSEN1* genes. Decline in olfactory function was observed in MC in the preclinical stages of AD, many years before the estimated clinical onset of disease. Decline was also observed in NC indicating that aging per se influences olfactory function. Olfactory function in MC and NC was related to episodic memory and to executive function. Odor identification may serve as a well-accepted, inexpensive, and valid test in clinical practice and research of AD.

## Data Availability

Data are available upon reasonable request and will be shared anonymized to a qualified academic investigator for replication of procedures and results presented in the article and if data transfer is in agreement with EU legislation on the general data protection regulation and decisions by the Ethical Review Board of Sweden. Sharing must be regulated in a material transfer agreement and or data processing agreement as appropriate.
